# Brain structural correlates of recurrence following the first episode in patients with major depressive disorder

**DOI:** 10.1038/s41398-022-02113-7

**Published:** 2022-08-27

**Authors:** Hannah Lemke, Hannah Klute, Jennifer Skupski, Katharina Thiel, Lena Waltemate, Alexandra Winter, Fabian Breuer, Susanne Meinert, Melissa Klug, Verena Enneking, Nils R. Winter, Dominik Grotegerd, Elisabeth J. Leehr, Jonathan Repple, Katharina Dohm, Nils Opel, Frederike Stein, Tina Meller, Katharina Brosch, Kai G. Ringwald, Julia-Katharina Pfarr, Florian Thomas-Odenthal, Tim Hahn, Axel Krug, Andreas Jansen, Walter Heindel, Igor Nenadić, Tilo Kircher, Udo Dannlowski

**Affiliations:** 1 Institute for Translational Psychiatry, University of Münster, Münster, Germany; 2 Institute for Translational Neuroscience, University of Münster, Münster, Germany; 3 Department of Psychiatry and Psychotherapy, University of Marburg, Marburg, Germany; 4 Department of Psychiatry and Psychotherapy, University of Bonn, Bonn, Germany; 5 University Clinic for Radiology, University of Münster, Münster, Germany

**Keywords:** Depression, Predictive markers

## Abstract

Former prospective studies showed that the occurrence of relapse in Major Depressive Disorder (MDD) is associated with volume loss in the insula, hippocampus and dorsolateral prefrontal cortex (DLPFC). However, these studies were confounded by the patient’s lifetime disease history, as the number of previous episodes predict future recurrence. In order to analyze neural correlates of recurrence irrespective of prior disease course, this study prospectively examined changes in brain structure in patients with first-episode depression (FED) over 2 years. *N* = 63 FED patients and *n* = 63 healthy controls (HC) underwent structural magnetic resonance imaging at baseline and after 2 years. According to their disease course during the follow-up interval, patients were grouped into *n* = 21 FED patients with recurrence (FEDrec) during follow-up and *n* = 42 FED patients with stable remission (FEDrem). Gray matter volume changes were analysed using group by time interaction analyses of covariance for the DLPFC, hippocampus and insula. Significant group by time interactions in the DLPFC and insula emerged. Pairwise comparisons showed that FEDrec had greater volume decline in the DLPFC and insula from baseline to follow-up compared with FEDrem and HC. No group by time interactions in the hippocampus were found. Cross-sectional analyses at baseline and follow-up revealed no differences between groups. This longitudinal study provides evidence for neural alterations in the DLPFC and insula related to a detrimental course in MDD. These effects of recurrence are already detectable at initial stages of MDD and seem to occur without any prior disease history, emphasizing the importance of early interventions preventing depressive recurrence.

## Introduction

Following the first episode, about 15-35% of patients with Major Depressive Disorder (MDD) develop recurrent episodes within the first years [1]. The number of lifetime episodes, severity of preceding episode and presence of subclinical residual symptoms have been stated as risk factors to suffer from additional recurrent episodes [2–4]—resulting in cumulative illness burden and chronicity in the long-term [5]. The investigation of neural correlates of recurrence in MDD may help to advance our understanding of the underlying pathological mechanisms, which could in future support the early identification of patients at high risk for an unfavorable disease course, and potentially improve treatments. There is a high need for longitudinal studies investigating the directionality of the associations between brain structure and disease course to clarify the current uncertainty of whether reduced brain volumes represent a cause or a consequence of recurrence. In this context, individuals with first-episode depression (FED) constitute a promising, well-characterized subgroup in which potentially confounding factors of prior disease history and treatment may have less impact on the underlying neural mechanisms of recurrence.

While cross-sectional research in MDD repeatedly showed that decreased gray matter volumes (GMV) and (sub-)cortical thickness are associated with more severe lifetime disease trajectories [6–10], the direct interplay between neural changes and recurrence remains unclear. Previous longitudinal studies reported morphometric changes [11], for example, in the dorsolateral prefrontal cortex (DLPFC), insula, hippocampus, and anterior cingulate cortex, showing greater GMV decline in dependence of more detrimental disease courses [12–16]. In this context, the occurrence of depressive relapse as a clearly distinguishable marker of disease progression has been specifically linked to GMV decline in cortical thickness and surface area in the insula and DLPFC [13, 14, 17]. Moreover, reductions of hippocampal GMVs in relation to chronic disease courses or residual symptomatology have been reported [12, 15, 16]. These findings clearly point toward adverse effects of disease progression on the morphology of reported brain regions. Nonetheless, other longitudinal studies did not find brain structural changes being related to disease trajectories [11, 18, 19]. Patients from all these prospective studies had experienced multiple episodes before study assessment [11], exacerbating the challenge to disentangle the distinct neural mechanisms of disease history and future recurrence.

In an initial longitudinal study on early depressive recurrence [20], the authors neither found longitudinal changes in FED patients suffering recurrence compared with FED patients remaining in full remission during the study interval, nor significant cross-sectional GMV differences between FED patients and HC at baseline and follow-up, respectively [20]. Notably, only a small sample of 27 FED patients and 17 HC was investigated. The results of this study stand in contrast to former literature reporting GMV alterations in FED patients compared with HC [21–24], and GMV changes associated with disease progression [8–16].

To overcome some of the above-mentioned challenging aspects regarding the longitudinal investigations of brain structure and disease course, the aim of this study was to prospectively investigate a larger sample of FED patients over a two-year span in order to enhance our understanding of potential neural risk factors for and consequences of recurrence without bias of disease history. To this end, we utilized data of voxel-based morphometry (VBM), cortical thickness, and surface area (Freesurfer, https://surfer.nmr.mgh.harvard.edu/) as such have already been investigated in relation to depressive recurrence [12–17, 25]. Based on literature reporting that the insula, hippocampus, and DLPFC seem to be particularly affected by the incidence of recurrence or disease progression [13, 14, 17], a ROI-approach was utilized. We expected that (a) FED patients with recurrence would exhibit more volume/thickness/surface decline compared with FED patients without recurrence and HC, (b) all FED patients would show less volume/thickness/surface compared with HC at baseline and follow-up, and, (c) baseline volume/thickness/surface differences between FED patients with and without recurrence may reveal a potential neural risk factors precipitating early recurrence.

## Materials and methods

### Participants and study design

For this study, participants from the Marburg-Münster-Affective-Cohort-Study (MACS) were selected. Baseline data were acquired between 2014/09 and 2016/09. Between 2016/10 and 2018/08, all participants were re-assessed about 2 years after their first study participation (mean = 2.10 years, SD = 0.16 years). Recruitment was conducted via psychiatric hospitals in Münster and Marburg, newspaper advertisement and flyers. General exclusion criteria comprised any history of neurological, autoimmune or cardiovascular disease, cancer, current pregnancy, head trauma, psychotic, schizoaffective and/or bipolar disorder, substance and alcohol dependence, intelligence quotient below 80 or common MRI contraindications (e.g. pacemakers, metal implants). Inclusion criteria for this study were age between 18 and 65 years at baseline and complete data of structural magnetic resonance imaging (MRI) and clinical interviews at both time points. The present study was independent of a previous 2-year follow-up study of our working group with no overlap in participants, scanners and imaging methods [13].

During face-to-face interviews, clinical diagnoses or the lack thereof were verified with the Structured Clinical Interview (SCID-I) according to DSM-IV-TR criteria [26] at both time points. These criteria resulted in a sample of *n* = 65 patients fulfilling criteria for a single (lifetime or current) episode of MDD at baseline. To enhance precision of each individual’s disease course, a life-chart method was utilized during follow-up interviews [27] to collect information about recurrence of depressive episodes, duration of depressive episodes and inpatient treatment during the study interval. Depending on the disease course during the interval, FED patients were divided into two subgroups: *n* = 21 patients experiencing at least one recurrent depressive episode (FEDrec) and *n* = 42 patients without subsequent episodes following the index episode and thus achieving or staying in remission (FEDrem). *N* = 2 FED patients experienced a chronic disease course during the follow-up interval and were therefore excluded as they could not be clearly assigned to one of the two patient groups. In order to fulfill criteria of discriminable recurrent illness episodes, patients had to remain symptom-free for at least two months before experiencing a new episode. In the case of recurrent depressive episodes, DSM-IV-TR criteria were applied to verify clinical pertinence. *N* = 63 healthy controls (HC), who did not fulfil any criteria of a lifetime or current psychiatric diagnosis were matched to the patients with respect to sex and age using the MatchIt package in R (Version 3.0.2, Table 1) [28].

**Table 1:** Sociodemographic and clinical characteristics of the total sample.

	FEDrec (*n* = 21)	FEDrem (*n* = 42)	*p*-Value^a^	HC (*n* = 63)	*p*-Value^b^
	mean ± SD	mean ± SD		mean ± SD	
**Baseline**					
Age	31.67 ± 12.93	38.79 ± 13.84	0.054^c^	36.48 ± 14.10	0.161^c^
Sex (f/m)	12/9	24/18	0.999^d^	39/24	0.296^d^
Verbal IQ_MWT-B_	111.00 ± 11.91	115.24 ± 13.59	0.230^c^	115.68 ± 14.69	0.398^c^
Scanner Settings (MR-BCpre, MR-BCpost, MS)	11/0/10	22/5/15	0.223^d^	35/1/27	0.114^d^
Smoking status (yes/no) (*n* = 125)	7/13	12/30	0.608^d^	7/56	0.023^d^
HDRS total score (*n* = 125)	8.40 ± 6.82	4.36 ± 4.51	0.034^e^	1.37 ± 1.99	<0.001^f^
Medication Load Index	1.33 ± 1.74	0.79 ± 1.07	0.116^e^	n/a	n/a
Psychiatric comorbidities (yes/no)	4/17	5/37	0.445^d^	n/a	n/a
CTQ total score	43.60 ± 11.83	40.40 ± 12.55	0.246^e^	31.95 ± 8.59	<0.001^f^
Familial risk for mood disorder (yes/no)	7/14	18/24	0.466^d^	8/55	0.002^d^
Remission status (acute/remitted)	14/7	19/23	0.108^d^	n/a	n/a
Time since first depressive symptoms (weeks)	63.43 ± 99.83	50.57 ± 63.08	0.605^e^	n/a	n/a
Duration of index episode (months) (*n* = 52)	13.68 ± 15.87	14.06 ± 13.24	0.960^e^	n/a	n/a
Psychiatric hospitalization at index episode (yes/no)	16/5	21/21	0.047^d^	n/a	n/a
**Follow-up**					
Smoking status (yes/no) (*n* = 120)	5/14	12/29	0.813^d^	8/52	0.125^d^
Body coil/site (BCpre, BCpost, Münster)	0/11/10	0/27/15	0.363^d^	0/36/27	0.622^d^
Interscan interval (months)	63.28 ± 3.12	65.04 ± 5.32	0.336^e^	63.34 ± 5.44	0.052^f^
HDRS total score	6.62 ± 5.90	2.55 ± 2.75	0.004^e^	1.25 ± 1.78	<0.001^f^
Medication Load Index	0.86 ± 1.11	0.36 ± 0.82	0.015^e^	n/a	n/a
Remission status (acute/remitted)	11/10	0/42	<0.001^d^	n/a	n/a
Number of depressive episodes during follow-up	1.48 ± 0.81	n/a	n/a	n/a	n/a
Duration of depressive episodes during follow-up (months)	6.49 ± 4.62	n/a	<0.001^e^	n/a	n/a
Rehospitalization during follow-up (yes/no)	9/12	0/42	<0.001^d^	n/a	n/a

*FEDrec* first-episode patients with recurrent episodes, *FEDrem* first-episode patients without recurrent episodes, *HC* healthy controls, *IQ*_*MWT-B*_ intelligence quotient according to the MWT-B, *BCpre* Body coil pre change, *BCpost* body coil post change, *HDRS* Hamilton Depression Ratings Scale, *CTQ* childhood trauma questionnaire.

^a^Comparison of patients with recurrent episodes and patients without recurrent episodes.

^b^Comparison of patients with recurrent episodes, patients without recurrent episodes and Healthy controls.

^c^One-way ANOVA or two-sample *t* test.

^d^χ^2^ test.

^e^Mann–Whitney *U* test.

^f^Kruskal–Wallis test.

At both time points, the presence and severity of depressive symptomatology was assessed with the Hamilton Depression Rating Scale (HDRS, [29]) and information on current psychopharmacological treatment was obtained. Psychopharmacological agents were combined into a medication load index, as previously utilized [30, 31]. To calculate the index, each active agent was coded according to the recommended average daily dose (0 = absent,1 = low/average dose, 2 = high dose) and summed up for each patient and each time point.

The study was conducted in accordance with the ethical guidelines and regulations of the Declaration of Helsinki and was approved by the Ethics Committees of the Medical Faculties of the Universities of Münster (AZ: 2014-422-b-S) and Marburg (AZ: 07/14). Prior to study participation, written informed consent was obtained and participants received financial compensation afterwards.

### Image acquisition

At both time points, T1-weighted high-resolution anatomical data were acquired at 3 T MRI scanners using three-dimensional (3D) fast gradient echo sequences (MPRAGE). Detailed information on acquisition parameters is provided in Supplement 1. Between baseline and follow-up, the body-coil at the Marburg site was exchanged, resulting in two dummy-coded variables (body-coil change yes vs. no) accounting for the scanner settings with Münster as reference category. Details of the quality assurance protocol and scanner harmonization of the present study can be found elsewhere [32].

### Pre-processing of VBM data

All T1-weighted images were pre-processed using the default settings of the longitudinal pipeline implemented in the CAT12-toolbox (http://www.neuro.uni-jena.de/cat,version r1742). This longitudinal VBM pipeline has been optimized to detect larger GMV changes over time intervals comprising several years. Pre-processing included the following steps using default parameters: Realignment, bias correction, tissue classification and spatial normalization to MNI-space were performed using the Geodesic Shooting algorithm. Additional modulation and warping steps were conducted as integrated into the longitudinal pre-processing pipeline of CAT12. Lastly, data were smoothed with an 8 mm full width at half-maximum Gaussian kernel. All images passed outlier detection of gray matter segmentation by using the check homogeneity function of CAT12 and visual inspections.

### Pre-processing of Freesurfer Data

The longitudinal pipeline of Freesurfer (Version 5.3.) was employed to pre-process all T1-weighted structural images. Following the standard protocol for cortical parcellation and subcortical segmentation of the ENIGMA consortium (http://enigma.ini.usc.edu/protocols/imaging-protocols), we used default parameters. Quality of resulting segments was assured by visual inspections and statistical evaluation of possible outliers, not resulting in the exclusion of data. Parcellation of cortical thickness and surface area was done using the Desikan-Killiany atlas [33], and segmentation of hippocampal volumes with the Aseg atlas [34].

### Statistical analyses

Descriptive and clinical data were analysed by using IBM SPSS Statistics 28 (SPSS Inc., Chicago, IL, USA). Statistical Parametric Mapping (SPM12, version 7771, Wellcome Department of Cognitive Neurology, London, UK) was used to analyze VBM data, while Freesurfer data were analysed using SPSS.

For all VBM second level analyses, the absolute threshold masking was set to 0.1 and threshold-free cluster enhancement (TFCE) implemented in the TFCE-toolbox (http://dbm.neuro.uni-jena.de/tfce,Version222) with 5000 permutations per test was applied. A statistical threshold using a family-wise-error (FWE) correction of *p* < 0.05 was employed. The DLPFC, hippocampus, and insula as regions of interest (ROI) were created as three separate bilateral masks. The DLPFC was defined according to Brodmann’s area 46 [35], and the insula and hippocampus according to the AAL-atlas definitions [36]. Both atlases are integrated into the WFU-pickatlas [37] in SPM12. All statistical analyses were performed separately for the three ROIs. For VBM analyses, extracted mean cluster values of significant clusters were used to calculate post-hoc pairwise comparisons in SPSS applying Bonferroni correction to account for multiple statistical tests (implying a threshold of *p* = 0.005 for 9 post-hoc *t*-tests: Three pairwise comparisons of groups at baseline, follow-up and within-group comparisons over time, respectively). Lastly, exploratory VBM whole-brain analyses were performed at a cluster threshold of *k* > 50 voxels, *p* < 0.001 uncorrected.

For Freesurfer analyses, right and left (sub-)cortical segments were used. The rostral middle frontal gyrus as a representative of the DLPFC [13], the insula and the hippocampus were chosen as ROIs. Again, all statistical analyses were performed for the ROIs separately and the Bonferroni method was applied for post-hoc *t*-tests.

#### Longitudinal analyses

For VBM data, *F*-tests on group by time interaction were analysed by calculating 3 × 2 analyses of covariance (ANCOVAs) including group (FEDrec, FEDrem, HC) as between-subjects factor and time (baseline, follow-up) as within-subjects factor using a second level flexible-factorial model in SPM12. A third factor “subject” was added to account for the individual constants of each participant. Age and scanner settings served as nuisance variables in the flexible-factorial models. Covariates, which do not change over time (such as sex) or underlie only subtle changes (such as total intracranial volume (TIV) [38]), are not recommended as control variables in flexible-factorial models by the CAT12 manual. The flexible-factorial model of SPM is particularly suitable for longitudinal analyses but does not allow meaningful cross-sectional contrasts. Therefore, the cross-sectional group analyses were performed for each time point in separate full-factorial ANCOVAs as described below.

For Freesurfer data, we performed 3 × 2 × 2-ANCOVAs for repeated measurements with group (FEDrec, FEDrem, HC) as between-subjects factor and time (baseline, follow-up) and hemisphere (right, left) as within-subjects factor in SPSS to calculate *F*-tests for the group by time interaction. Analyses of DLPFC and insula thickness included the covariates age, sex and scanner settings, and analyses of surface area were additionally controlled for TIV. For hippocampal volumes, the covariates were age, sex, scanner settings, and TIV.

#### Cross-sectional analyses

To investigate cross-sectional group effects and to identify potential neural risk factors for recurrence, we performed full-factorial ANCOVAs for VBM data with (FEDrec, FEDrem, HC) as between-subjects factor controlling for age, sex, TIV, and scanner settings at baseline and follow-up, separately.

For the cross-sectional analyses of Freesurfer data, we performed separate 3 × 2 ANCOVAs with a group (FEDre, FEDrem, HC) as between-subjects factor and hemisphere (right, left) as within-subjects-factor for baseline and follow-up, respectively. Included control variables were again age, sex, and scanner settings, and additionally TIV for surface area and hippocampal volumes.

#### Additional analyses

In case of the significant group by time effects in the main ANCOVAs of VBM and Freesurfer data, and in order to control for potentially confounding effects of pharmacotherapy, current symptomatology, and remissions status, we performed further 2 × 2 ANCOVAs for VBM and 2 × 2 × 2 ANCOVAs for Freesurfer data by (1) including only FED patients while additionally controlling for medication and HDRS scores, and (2) including only acutely depressed patients at baseline (FEDrec: *n* = 14, FEDrem: *n* = 19) while controlling for medication. All additional analyses included the previously described control variables (age, sex, scanner settings, and TIV in the case of Freesurfer surface area or hippocampal volumes).

## Results

### Descriptive and clinical characteristics

The groups did not differ significantly regarding age, sex, and site (Table 1). FED patient groups differed significantly in HDRS scores at baseline as well as HDRS scores, remission status and medication at follow-up. For details on medication and the presence of psychiatric comorbidities, see Supplement 2.

### Longitudinal analyses

#### DLPFC

**Fig. 1:**
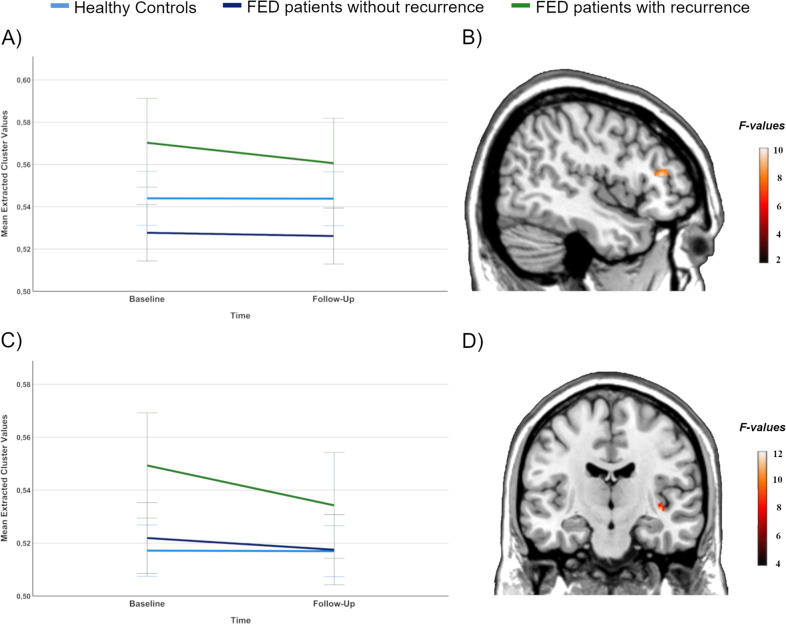
Group by time interaction of the DLPFC and insula. **A** Mean cluster values of the group by time interaction of the right dorsolateral prefrontal cortex for healthy controls, FED patients without recurrence and FED patients with recurrence. **B** Significant cluster of the group by time interaction in the right dorsolateral prefrontal cortex depicted at *x* = 46, *y* = 34, z = 14, with a threshold of *p* < 0.001, uncorrected. **C** Mean cluster values of the group by time interaction of the right insula for healthy controls, FED patients without recurrence and FED patients with recurrence. **D** Significant cluster of the group by time interaction in the right insula depicted at *x* = 39, *y* = −15, *z* = −6 with a threshold of *p* < 0.001, uncorrected. Error bars indicate one standard error.

In the 3 × 2-ANCOVA of VBM data, a significant group by time interaction in the right DLPFC emerged (*F*(2,121) = 9.26, *p*_tfce-FWE_ = 0.007, *k* = 230 voxels, *partial η²* = 0.107, Fig. 1A, B). No significant group by time interaction was found in the left DLPFC (*p*_tfce-FWE_ = 0.053). Post-hoc *t*-tests revealed that FEDrec patients showed significant volume decline in the right DLPFC from baseline to follow-up (*t*(20) = 5.06, *p* < 0.001, *95%-CI:* 0.550 to 1.642, *Cohen’s d* = 1.11) while no significant volume decline was found in FEDrem patients (*p* = 0.305) and HC (*p* = 0.881).

The 3 × 2 × 2-ANCOVA of DLPFC thickness data revealed a significant group by time interaction (*F*(2,118) = 4.77, *p* = 0.010, *partial η²* = 0.075, Supplement 3). In line with the foregoing VBM results, FEDrec patients showed a significant decline in bilateral DLPFC thickness from baseline to follow-up (right: *t*(20) = 4.75, *p* < 0.001, Cohen’s *d* = 1.04, *mean difference*: 0.0365 mm, *95%-CI*: 0.0205 to 0.0526 mm; left: *t* (20) = 2.73, *p* = 0.013, Cohen’s *d* = 0.59, *mean difference*: 0.0361 mm, *95%-CI*: 0.0085 to 0.0636 mm). DLPFC volumes did not change over time in HC (right: *p* = 0.718; left: *p* = 0.631) and FEDrem patients (right: *p* = 0.977; left: *p* = 0.352). When repeating the analysis separately for both hemispheres, significant group by time interactions were found for right (*F*(2,118) = 3.46, *p* = 0.035, partial *η²* = 0.055) and left DLPFC thickness (*F*(2,118) = 3.61, *p* = 0.030, partial *η²* = 0.058).

The 3 × 2 × 2-ANCOVA of DLPFC surface area did not reveal a significant group by time interaction (*p* = 0.102).

#### Insula

The 3 × 2-ANCOVA of VBM data showed a significant group by time interaction in the right insula (*F*(2,121) = 11.84, *p*_tfce-FWE_ = 0.036, *k* = 13 voxels, *partial η*² = 0.129, Fig. 1C, D). No significant group by time interaction effect was found in the left insula (*p*_tfce-FWE_ = 0.204). Post-hoc *t*-tests indicated that right insula volumes decreased significantly from baseline to follow-up in FEDrec patients (*t*(20) = 6.77, *p* < 0.001, Cohen’s *d* = 1.47). In the FEDrem group, insula volume tended to decrease (*p* = 0.024), however, not statistically significant after Bonferroni correction. For HC, no significant insula volume decline from baseline to follow-up was found (*p* = 0.898).

The Freesurfer analyses neither revealed a significant group by time interaction in the 3 × 2 × 2-ANCOVA of insula thickness (*p* = 0.730) nor surface (*p* = 0.922).

#### Hippocampus

Neither the 3 × 2-ANCOVA of VBM data (*p*_tfce-FWE_ = 0.309) nor the 3 × 2 × 2-ANCOVA of Freesurfer data (*p* = 0.391) showed a significant group by time interaction in hippocampal volumes.

#### Whole-brain

The exploratory VBM whole-brain analysis of the group by time interaction showed decreased GMVs in the insula, precuneus, (orbito-)frontal and temporal regions in FEDrec patients compared with HC and in temporal and frontal regions in FEDrec compared with FEDrem patients over the two year follow-up period (Supplement 4).

### Cross-sectional analyses

#### DLPFC

In the cross-sectional analyses of DLPFC GMVs, neither a main effect of group at baseline (*p*_tfce-FWE_ = 0.164) nor at follow-up (*p*_tfce-FWE_ = 0.354) was found. Further, the analyses of DLPFC thickness and surface data revealed no main effects of group at baseline (thickness: *p* = 0.400; surface: *p* = 0.185) and follow-up (thickness: *p* = 0.998; surface: *p* = 0.115).

#### Insula

No significant group effects at baseline (*p*_tfce-FWE_ = 0.784) and follow-up (*p*_tfce-FWE_ = 0.336) were found for insula GMVs in VBM data. The analyses of insula thickness and surface area also revealed no main effect of group at baseline (thickness: *p* = 0.749; surface: *p* = 0.341) nor at follow-up (thickness: *p* = 0.945; surface: *p* = 0.209).

#### Hippocampus

The cross-sectional analyses of hippocampal volumes neither revealed a group effect at baseline (*p*_tfce-FWE_ = 0.999) nor at follow-up (*p*_tfce-FWE_ = 0.999) in VBM data. Freesurfer data also showed no group differences in hippocampal volumes at baseline (*p* = 0.068) and follow-up (*p* = 0.085).

#### Whole-brain

The exploratory VBM whole-brain analysis at baseline revealed lower GMV in temporal regions in FEDrec patients compared with HC, parietal and temporal regions in FEDrem patients compared with HC and frontal, parietal, and temporal regions in FEDrem compared with FEDrec patients (Supplement 5). At follow-up, the analysis showed decreased GMV in temporal regions and caudate nucleus in FEDrec patients compared with HC, parietal and temporal regions in FEDrem patients compared with HC and frontal, temporal, and parietal regions in FEDrem compared with FEDrec patients (Supplement 6).

### Additional analyses

#### DLPFC

In the 2 × 2-ANCOVAs, the group by time interaction remained bilaterally significant when (1) additionally controlling for psychiatric medication and HDRS scores (right: *F*(1,57) = 14.81, *p*_tfce-FWE_ = 0.019, *k* = 160 voxels, *partial η*^2^ = 0.132; left: *F*(1,57) = 11.14, *p*_tfce-FWE_ = 0.039, *k* = 6 voxels, partial *η*^2^ = 0.094), and (2) only including acutely depressed patients (right: *F*(1,28) = 15.55, *p*_tfce-FWE_ = 0.037, *k* = 72 voxels, *partial η*^2^ = 0.277; left: *F*(1,28) = 15.20, *p*_tfce-FWE_ = 0.029, *k* = 25 voxels, *partial η*^2^ = 0.175).

Repeating the 2 × 2 × 2-ANCOVA with DLPFC thickness, the group by time interaction remained significant when (1) additionally controlling for medication and HDRS scores (*F*(1,51) = 7.69, *p* = 0.008, *partial η*^2^ = 0.131). Looking at lateralization effects, significant group by time interactions were found for right (*F*(1,52) = 5.75, *p* = 0.020, partial *η*^2^ = 0.100) and left DLPFC thickness (*F*(1,52) = 4.55, *p* = 0.038, partial *η*^2^ = 0.081), after repeating the analyses for both hemispheres separately. For the 2 × 2 × 2 ANCOVA including (2) only acutely depressed FED patients, the group by time interaction was also found (*F*(1,24) = 4.79, *p* = 0.039, *partial η*^2^ = 0.167). This effect was located in right (*F*(1,25) = 4.96, *p* = 0.035, *partial η*^2^ = 0.166), but not detected in left DLPFC thickness (*p* = 0.111).

#### Insula

Neither the 2 × 2-ANCOVA of insula GMVs including only FED patients under control of medication and HDRS scores (*p*_tfce-FWE_ = 0.117), nor the analyses including only acutely depressed patients (*p*_tfce-FWE_ = 0.053) reached statistical significance.

The 2 × 2 × 2-ANCOVA of insula thickness neither revealed a significant group by time interaction between FED groups (*p* = 0.521) controlling for HDRs scores and medication, nor in the analyses including acutely depressed FED patients only (*p* = 0.162).

## Discussion

This study investigated longitudinal changes as well as cross-sectional differences regarding brain structure in patients with first-episode depression in a prospective design over 2 years. Our results show that FED patients with recurrence following the index episode have more volume decline in DLPFC volumes and thickness and insula volumes than HC and FED patients in remission. The brain structural changes in the DLPFC were not affected by pharmacotherapy, current symptomatology and patient’s remission status at baseline and follow-up. In the cross-sectional analyses, we neither found significant differences between groups in DLPFC and insula GMV, thickness or surface at baseline nor at follow-up. For hippocampal volumes, the results showed no significant group by time interaction over the 2 years, and no significant cross-sectional group differences.

Our results of volume and thickness decline of the DLPFC as a consequence of recurrence are in line with a previous study [13]. Further, the additional analyses controlling for pharmacological treatment and current depression severity suggest that volume/thickness decline of the DLPFC is rather associated with recurrent disease course than other clinical characteristics. The DLPFC has been repeatedly implicated in the pathophysiology of MDD [39] and involved in executive operations such as decision making, goal-directed behavior and emotion regulation [40, 41]. Animal and (postmortem) human studies suggest stress-induced neurotoxic effects in the prefrontal brain of MDD patients [42] and that these alterations aggravate with disease progression [13], as also in line with our findings. Moreover, treatment studies imply involvement of the DLPFC in the recovery of MDD, as repetitive transcranial magnetic stimulation of the DLPFC can lead to increased volumes of the insula, anterior cingulate cortex, temporal and angular gyrus [43]. Together with previous literature, our results underline the importance of the DLPFC in the progression of MDD—already being critically involved at an early stage.

Furthermore, we found insula volume decline in dependence of recurrence during the study interval, but no significant effects for insula thickness. The insula has also been implicated in the pathophysiology of MDD [44] and has a broad range of functions including the integration of interoceptive information about the person’s own emotional state, salience detection and empathic experience and is reciprocally connected with the DLPFC [45]. The finding, that insula volumes are affected by recurrence is in line with prior longitudinal studies reporting lower insula GMV and thickness being associated with depressive recurrences [13, 14]. However, after the additional control of medication, current symptomatology and remission states, the effect was no longer significant. Furthermore, insula thickness and surface measures were not associated with depressive recurrence in our study, which is contrary to a former study reporting insula thickness decline following depressive relapse in MDD [13]. Together, these findings cannot provide a clear role of the insula in MDD disease course and effects could possibly be attributed to time effects rather than specific disease trajectories, especially in the early phase of disease. This could explain why patients without recurrence also showed slight decline of insula GMVs, although this result did not survive Bonferroni correction.

Regarding hippocampal structure, we did not find longitudinal effects of recurrence, standing in contrast to previous prospective MRI studies [16, 46–48]. One difference to our study and potentially explaining the disparity is that MDD patients from previous reports were diagnosed with MDD several years before baseline assessment and most of them had already suffered from multiple lifetime episodes. Thus, morphometric alterations in the hippocampus may be more likely observable in individuals after prolonged illness. In line, two meta-analyses did not report alterations in hippocampal GMVs in FED patients compared with HC, and the authors pointed towards accumulating neurotoxic effects on hippocampal structure in prolonged but not early disease course [49, 50]. In corroboration, a recent review suggests an essential role of chronic stress in the development of hippocampal pathology [51]. Concluding, hippocampal pathology may only develop over disease progression and may not be detectable at the beginning of disease.

The exploratory GMV whole-brain results suggest that furthermore frontal and temporal brain regions and the putamen show GMV reductions in dependence of recurrence. In conjunction with previous longitudinal studies [12–16, 52], our findings point towards more widespread effects of long-term disease course on brain structure. Nonetheless, our exploratory whole-brain results need to be interpreted with caution due to the uncorrected threshold.

In the cross-sectional analyses, we neither detected volume, thickness nor surface alterations in the insula, hippocampus and DLPFC as neural markers for depression at baseline and follow-up. These results stand in contrast to former meta-analyses which reported brain structural differences in several regions including the prefrontal brain, hippocampus and insula in MDD patients compared with HC [21, 22, 53–56]. Thus, it seems surprising that we did not find cross-sectional group differences in the ROI analyses in our study. However, former brain structural effects were more consistently found in patients with recurrent than in first-episode MDD [21]. Moreover, previous meta-analyses present small to moderate effects (*d* = 0.12 to *d* = 0.57 [21, 53, 54, 56]), which could not be detected in our comparably small sample primarily designed to detect longitudinal effects and not to replicate cross-sectional differences. In line with our results, another study also did not report cross-sectional group differences between HC and FED patients in a small study sample [20]. G*Power (version 3.1) calculates *n* > 60 participants per group to detect moderate effect sizes of *d* ≥ 0.025 [57], emphasizing the need for larger sample sizes in follow-up studies. Nevertheless, our exploratory whole-brain analyses point towards neural alterations more likely located in temporal and parietal regions in early disease patients.

The cross-sectional results also did not point towards the DLPFC, hippocampus and insula as putative neural risk markers predicting later recurrence. While attention has been paid to fronto-limbic regions as potential biomarkers being involved in the progression of MDD [58], consistent results are still missing, possibly owing to small sample sizes and distinct operationalization of disease trajectories [59]. Presuming high clinical relevance, the contribution of neurobiological markers supporting the early identification of patients at high risk for recurrence need further investigation. In this context, the employment of multivariate machine-learning techniques may be more promising approaches [59–61].

The major strength of our study is that FED patients comprise a well-characterized subgroup of MDD patients. The longitudinal investigation of patient’s disease course following the initial illness episode may help to extend current knowledge on neural mechanisms related to recurrence in MDD, simultaneously minimizing confounding effects of prior disease and treatment history. Another strength is that we performed additional analyses by 1) controlling for pharmacotherapy and symptom severity at both time points to investigate the potential influence of treatment and disease related characteristics, and, 2) repeated the analyses by only including acutely depressed patients to investigate whether recurrence effects remain stable when patient groups have equal remission states at baseline. Third, we utilized two different techniques to analyze measures of brain structure and could partly replicate the GMV results in thickness values supporting the robustness of our findings.

Some limitations need to be addressed. First, FED patients were either currently experiencing their first episode or had already experienced their first episode in the past and were therefore already remitted at baseline. This circumstance differs from former longitudinal studies which mainly included homogeneous patient groups with HDRS cut-offs or equal affective states at baseline [13, 16]. To account for this sample heterogeneity, we performed additional analyses for remission status. Second, FEDrec patients showed more severe depressive symptoms at baseline, reflected by higher HDRS scores. To account for this difference, we included HDRS scores as control variable in the additional analyses. Noteworthy, this incident may contribute to the naturalistic classification of our sample based on disease course, as one important predictor for recurrence is the severity of the preceding episode [2, 4, 5]. Third, at baseline a higher proportion of FEDrec patients were under pharmacotherapy compared to FEDrem patients (FEDrec:71%; FEDrem:42%; Supplement 2). While we controlled for psychopharmacological intake to control for potential effects on brain structure, this might have further subtracted out some variance underlying our classification of patients groups according to naturalistic disease course. Fourth, although our sample size was larger compared to the initial study investigating brain structural correlates of recurrence in FED patients [20], we strongly encourage future studies to validate these findings in larger datasets. Lastly, we only gathered information about psychiatric agents at both time points, but did not collect data on psychiatric agents during follow-up. Therefore, our recurrence-associated effects could have been affected by adjustment of medication during follow-up.

In conclusion, this study provides preliminary evidence that recurrence at an early stage of MDD can lead to detrimental effects in brain regions that are involved in emotion perception and regulation [40, 44, 45, 62, 63]. Although alterations of the DLPFC, hippocampus and insula were not identified as neural precursors of recurrence at this initial illness stage, the DLPFC and insula seem particularly sensitive to neurotoxic effects of recurrence over time and may therefore already play a crucial role in the early progression of MDD. To further elucidate consequential versus precipitating neural effects of depressive recurrence, larger samples of FED patients combining multivariate approaches and allowing sub-analyses considering remission states, symptom severity or comparing drug-naïve with psychopharmacologically treated individuals are encouraged.

## Supplementary information

Supplementary Material
