# Supplementary material for: Brain structural correlates of recurrence following the first episode in patients with major depressive disorder
Source: Transl Psychiatry. 2022 Aug 27;12:349. doi: 10.1038/s41398-022-02113-7 (PMC9420111; doi:10.1038/s41398-022-02113-7)
Supplement: Supplementary file 1 — Supplementary Material [file 41398_2022_2113_MOESM1_ESM.docx]

**Supplementary Information**

**Supplement 1: MRI Acquisition Parameters**

At baseline and follow-up, T1-weighted high-resolution anatomical data were acquired at 3T whole body MRI scanners using three-dimensional (3D) fast gradient echo sequences (MPRAGE) including the following parameters. Münster: Prisma (Siemens, Erlangen, Germany), 192 sagittal slices, TR=2130ms, TE=2.28ms, inversion time=900ms, FA=8°, resulting in a voxel size of 1x1x1mm³. Marburg: Tim Trio (Siemens, Erlangen, Germany), 176 sagittal slices, TR=1900ms, TE=2.26ms, inversion time=900ms, FA=9°, resulting in a voxel size of 1x1x1mm³.

**Supplement 2: Table with Information on Psychopharmacological Intake and Psychiatric Comorbidities in FED patients**

*Information on psychopharmacological intake of FED patients at baseline and follow-up.*

|  | **FEDrec**  **(n=21)** | **FEDrem**  **(n=42)** |
| --- | --- | --- |
|  | mean ± SD | mean ± SD |
| **Baseline** |  |  |
| Medication Load Index | 1.33 ± 1.74 | 0.79 ± 1.07 |
| None | 6 | 24 |
| SSRI^1^ | 8 | 5 |
| SNRI^1^ | 6 | 7 |
| NDRI/NARI^1^ | 0 | 0 |
| NaSSA^1^ | 2 | 4 |
| Tricyclic antidepressants^1^ | 0 | 2 |
| MAO inhibitors^1^ | 0 | 0 |
| Antipsychotics^1^ | 3 | 4 |
| Others^1^ | 4 | 4 |
| Psychiatric comorbidities (yes/no) | 3/18 | 5/37 |
| Social Anxiety Disorder^1^ | 2 | 1 |
| Specific Phobia^1^ | 0 | 3 |
| Panic Disorder^1^ | 0 | 1 |
| Generalized Anxiety Disorder^1^ | 0 | 0 |
| Obsessive-Compulsive Disorder^1^ | 0 | 0 |
| Eating Disorder^1^ | 1 | 0 |
| Posttraumatic Stress Disorder^1^ | 0 | 1 |
| Other^1^ | 1 | 2 |
| **Follow-up** |  |  |
| Medication Load Index | 0.86 ± 1.11 | 0.36 ± 0.82 |
| None | 10 | 33 |
| SSRI^1^ | 4 | 2 |
| SNRI^1^ | 4 | 5 |
| NDRI/NARI^1^ | 2 | 0 |
| NaSSA^1^ | 0 | 0 |
| Tricyclic antidepressants^1^ | 0 | 2 |
| MAO inhibitors^1^ | 0 | 0 |
| Antipsychotics^1^ | 1 | 2 |
| Others^1^ | 3 | 1 |
| Psychiatric comorbidities (yes/no) | 3/18 | 4/38 |
| Social Anxiety Disorder^1^ | 1 | 1 |
| Specific Phobia^1^ | 0 | 2 |
| Panic Disorder^1^ | 0 | 1 |
| Generalized Anxiety Disorder^1^ | 0 | 0 |
| Obsessive-Compulsive Disorder^1^ | 0 | 0 |
| Eating Disorder^1^ | 1 | 1 |
| Posttraumatic Stress Disorder^1^ | 0 | 0 |
| Other^1^ | 1 | 3 |

Abbreviations. FEDrec=first-episode patients with recurrent episodes, FEDrem=first-episode patients without recurrent episodes, SSRI=Selective Serotonin Reuptake Inhibitor, SNRI= Serotonin-Norepinephrine Reuptake Inhibitors, NDRI/NaRI= Norepinephrine–Dopamine Reuptake Inhibitor/ Noradrenaline Reuptake Inhibitor, NaSSA= Noradrenergic and Specific Serotonergic Antidepressant.

**Supplement 3: Figure of the Results of the 3x2x2-ANCOVA of DLPFC Thickness Values**


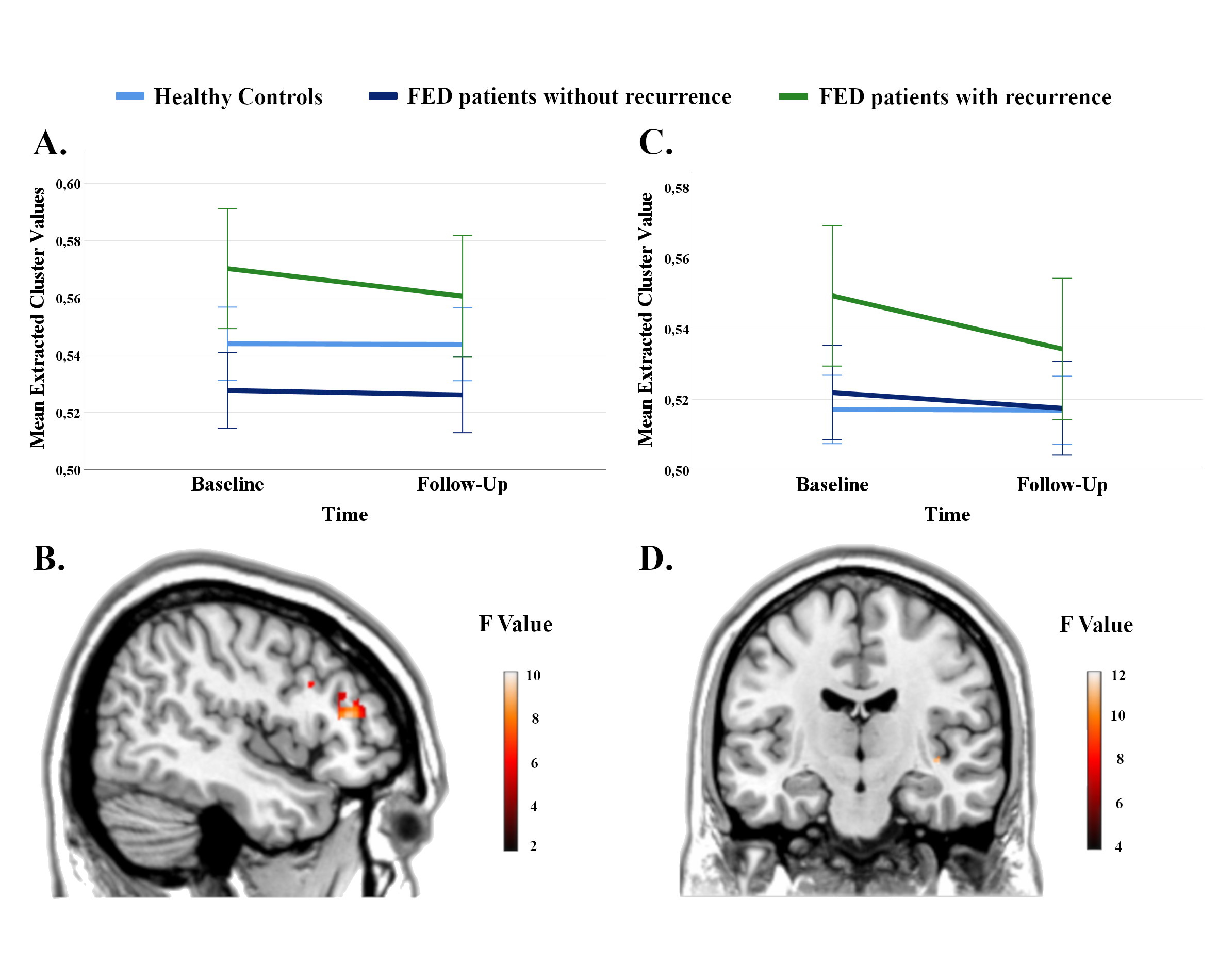

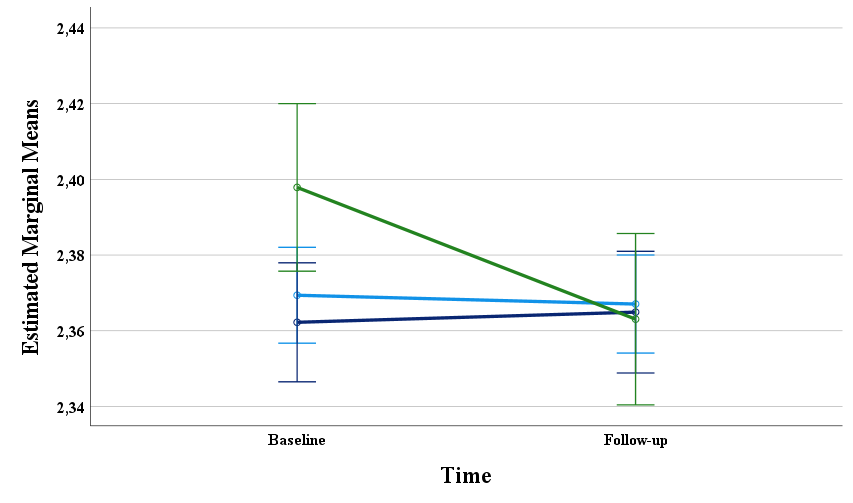


**Figure 1.S3.** Freesurfer thickness values of the group by time interaction of the bilaterally averaged dorsolateral prefrontal cortex for healthy controls, FED patients without recurrence and FED patients with recurrence. Error bars indicate one standard error.

**Supplement 4: Table of the Exploratory Whole-Brain Results for the Group by Time Interaction**

*Results of group by time interaction in the exploratory whole-brain analyses conducted at p<.001, uncorrected, with a threshold of k=50.*

| **Anatomic Label** | **Hemisphere** | **Cluster Size** | **x** | **y** | **z** | ***F/t*-Score** |
| --- | --- | --- | --- | --- | --- | --- |
| *Contrast: F-test Group by time interaction* | | | | | | |
| Cerebelum, crus 8 and 9 | R | 1363 | 2 | -62 | -46 | 12.49 |
| Insula, putamen | R | 182 | 38 | -15 | -8 | 12.07 |
| Precuneus | R | 75 | 10 | -63 | 42 | 10.45 |
| Middle frontal gyrus | L | 53 | -40 | 54 | 16 | 10.26 |
| Anterior and lateral orbitofrontal cortex | R | 84 | 39 | 45 | -15 | 9.60 |
| Middle frontal gyrus | R | 117 | 38 | 58 | 16 | 9.26 |
| Inferior frontal gyrus | R | 109 | 46 | 34 | 14 | 9.26 |
| Postcentral and precentral gyrus | R | 105 | 50 | -14 | 45 | 9.19 |
| *Contrast: HC follow-up>baseline versus FEDrem baseline>follow-up* | | | | | | |
| Postcentral gyrus | L | 63 | -22 | -33 | 56 | 3.99 |
| Ventral striatum, olfactory gyrus | R | 272 | 10 | 0 | -6 | 3.84 |
| Postcentral gyrus | L | 55 | -38 | -21 | 50 | 3.65 |
| *Contrast: HC follow-up>baseline versus FEDrec baseline>follow-up* | | | | | | |
| Insula, putamen | R | 553 | 38 | -15 | -8 | 4.85 |
| Inferior temporal gyrus | R | 161 | 40 | 12 | -48 | 4.58 |
| Precuneus | R | 288 | 10 | -63 | 42 | 4.55 |
| Middle frontal gyrus | L | 149 | -40 | 54 | 16 | 4.45 |
| Lateral and anterior orbitofrontal cortex | R | 491 | 39 | 45 | -15 | 4.34 |
| Cerebellum, crus 8 | R | 415 | 20 | -56 | -57 | 4.29 |
| Inferior and middle frontal gyrus | R | 2141 | 38 | 58 | 18 | 4.28 |
| Postcentral and precentral gyrus | R | 343 | 57 | -21 | 45 | 4.09 |
| Inferior and middle frontal gyrus | L | 532 | -46 | 21 | 32 | 4.09 |
| Caudate nucleus | L | 253 | -12 | 3 | 18 | 4.04 |
| Superior frontal gyrus | R | 262 | 16 | 57 | 3 | 3.99 |
| Fusiform gyrus | L | 79 | -36 | -16 | -28 | 3.96 |
| Precuneus | L | 324 | -9 | -62 | 48 | 3.94 |
| Superior temporal gyrus | L | 58 | -66 | -50 | 21 | 3.78 |
| Precentral gyrus | L | 79 | -58 | 4 | 30 | 3.65 |
| Superior frontal gyrus | R | 342 | 24 | 18 | 48 | 3.61 |
| Rolandic operculum | R | 65 | 56 | 6 | 9 | 3.57 |
| *Contrast: FEDrem follow-up>baseline versus FEDrec baseline>follow-up* | | | | | | |
| Supramarginal gyrus, postcentral gyrus | R | 155 | 58 | -22 | 45 | 3.95 |
| Superior temporal gyrus | L | 72 | -64 | -50 | 21 | 3.94 |
| Posterior cingulate cortex | R | 82 | 2 | -42 | 38 | 3.94 |
| Inferior frontal gyrus | L | 147 | -56 | 22 | 21 | 3.93 |
| Middle frontal gyrus | R | 218 | 50 | 16 | 42 | 3.86 |
| Middle frontal gyrus | R | 134 | 34 | 30 | 34 | 3.80 |
| Inferior frontal gyrus | R | 157 | 46 | 36 | 12 | 3.80 |
| Middle frontal gyrus | L | 94 | -40 | 54 | 16 | 3.80 |
| Superior frontal gyrus | L | 165 | -16 | 32 | 46 | 3.76 |
| Angular gyrus | L | 196 | -40 | -60 | 33 | 3.61 |
| Insula | L | 53 | -30 | 21 | 10 | 3.54 |
| Superior frontal gyrus | L | 76 | -12 | 54 | 44 | 3.40 |

*Abbreviations:* L=Left, R=Right, FEDrec=First episode patients with recurrence depression, FEDrem=First episode patients without recurrence.

**Supplement 5: Table of the Exploratory Whole-Brain Results for the Main Effect of Group at Baseline**

*Results of baseline main effects of group in the exploratory whole-brain analyses conducted at p<.001, uncorrected, with a threshold of k=50.*

| **Anatomic Label** | **Hemisphere** | **Cluster Size** | **x** | **y** | **z** | ***F/t*-Score** |
| --- | --- | --- | --- | --- | --- | --- |
| Contrast: *F*-Test |  |  |  |  |  |  |
| Inferior and middle temporal gyrus | L | 64 | -39 | -57 | -6 | 10.86 |
| Inferior parietal gyrus | L | 71 | -39 | -57 | 51 | 8.54 |
| Contrast: HC > FEDrem |  |  |  |  |  |  |
| Superior parietal gyrus, angular gyrus | R | 312 | 30 | -68 | 48 | 3.96 |
| Inferior parietal gyrus | L | 335 | -39 | -57 | 51 | 3.76 |
| Inferior and middle temporal gyrus | L | 53 | -39 | -57 | -6 | 3.69 |
| Middle temporal gyrus | L | 92 | -63 | -60 | 8 | 3.52 |
| Contrast: HC > FEDrec |  |  |  |  |  |  |
| Inferior and middle temporal gyrus | L | 59 | -36 | -56 | -8 | 3.92 |
| Contrast: FEDrem > FEDrec |  |  |  |  |  |  |
|  | - | - | - | - | - | - |
| Contrast: FEDrec > FEDrem |  |  |  |  |  |  |
| Inferior frontal gyrus | R | 321 | 45 | 4 | 21 | 3.95 |
| Inferior parietal gyrus | L | 235 | -42 | -48 | 38 | 3.90 |
| Superior temporal gyrus | R | 375 | 68 | -38 | 22 | 3.86 |
| Postcentral gyrus | R | 66 | 52 | -24 | 52 | 3.64 |
| Precuneus | L | 74 | -8 | -64 | 58 | 3.51 |

*Abbreviations:* L=Left, R=Right, HC=Healthy Controls, FEDrec=First episode patients with recurrence depression, FEDrem=First episode patients without recurrence.

**Supplement 6: Table of the Exploratory Whole-Brain Results for the Main Effect of Group at Follow-Up**

*Results of follow-up main effects of group in the exploratory whole-brain analyses conducted at p<.001, uncorrected, with a threshold of k=50.*

| **Anatomic Label** | **Hemisphere** | **Cluster Size** | **x** | **y** | **z** | ***F/t*-Score** |
| --- | --- | --- | --- | --- | --- | --- |
| *Contrast: F-test* |  |  |  |  |  |  |
| Superior parietal gyrus | R | 117 | 34 | -66 | 57 | 10.13 |
| Middle temporal gyrus | L | 85 | -63 | -60 | 6 | 9.12 |
| *Contrast: HC > FEDrem* |  |  |  |  |  |  |
| Superior parietal gyrus, angular gyrus | R | 396 | 34 | -66 | 57 | 4.42 |
| Middle temporal gyrus | L | 155 | -62 | -60 | 14 | 3.99 |
| Inferior and superior parietal gyrus | L | 246 | -38 | -58 | 52 | 3.61 |
| *Contrast: HC > FEDrec* |  |  |  |  |  |  |
| Inferior temporal gyrus, fusiform gyrus | L | 75 | -38 | -54 | -8 | 4.05 |
| Caudate nucleus | L | 128 | -6 | 15 | 12 | 3.74 |
| Middle temporal gyrus | R | 89 | 62 | -52 | 2 | 3.41 |
| *Contrast: FEDrem > FEDrec* |  |  |  |  |  |  |
| Putamen | R | 66 | 27 | 16 | 6 | 3.44 |
| *Contrast: FEDrec > FEDrem* |  |  |  |  |  |  |
| Inferior frontal gyrus | R | 264 | 45 | 4 | 21 | 3.88 |
| Superior temporal gyrus | R | 185 | 68 | -38 | 22 | 3.63 |
| Inferior parietal gyrus | L | 52 | -42 | -48 | 38 | 3.51 |

*Abbreviations:* L=Left, R=Right, FEDrec=First episode patients with recurrence depression, FEDrem=First episode patients without recurrence.
